# Supplementary material for: Directed evolution of aminoacyl-tRNA synthetases through in vivo hypermutation
Source: Nat Commun. 2025 May 24;16:4832. doi: 10.1038/s41467-025-60120-w (PMC12103617; doi:10.1038/s41467-025-60120-w)
Supplement: Supplementary file 1 — Supplementary Information [file 41467_2025_60120_MOESM1_ESM.pdf]

Supplementary Information for:

Directed evolution of aminoacyl-tRNA synthetases through *in vivo* hypermutation

Yuichi Furuhata[1,2,3], Gordon Rix[3,4], James A. Van Deventer[5,6], and Chang C. Liu[1,3,4,7,*]

[1]Department of Biomedical Engineering, University of California, Irvine, CA, USA

[2]Molecular Biosystems Research Institute, National Institute of Advanced Industrial Science and Technology (AIST), Tsukuba, Ibaraki, Japan

[3]Center for Synthetic Biology, University of California, Irvine, CA, USA

[4]Department of Molecular Biology and Biochemistry, University of California, Irvine, CA, USA

[5]Department of Chemical and Biological Engineering, Tufts University, Medford, MA, USA

[6]Department of Biomedical Engineering, Tufts University, Medford, MA, USA

[7]Department of Chemistry, University of California, Irvine, CA, USA

[*]Corresponding author. E-mail: ccl@uci.edu

Supplementary Figures

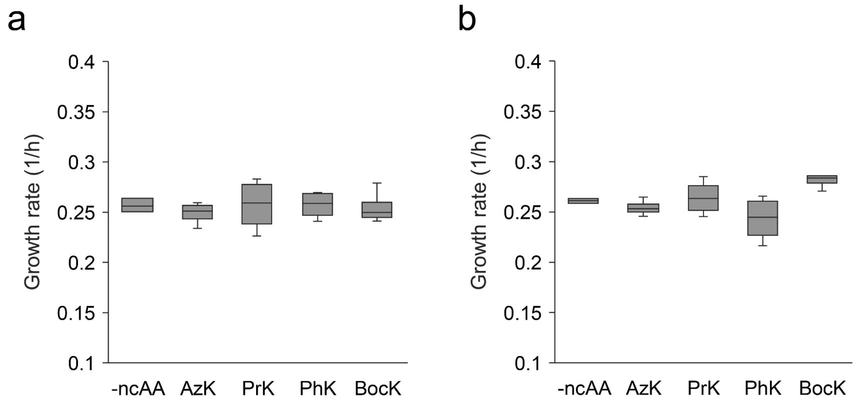

Supplementary Fig. 1. Evaluation of amber codon suppression on yeast growth. (a, b) Growth rate of yeast strains expressing amber suppressor *Ma*tRNA^{Pyl} with an empty vector (a) and *Ma*PylRS/PhK-A (b) was measured with or without 1 mM ncAA. Each condition was measured in technical quadruplicates. The median, first and third quartiles, minimum and maximum are depicted. Source data are provided as a Source Data file.

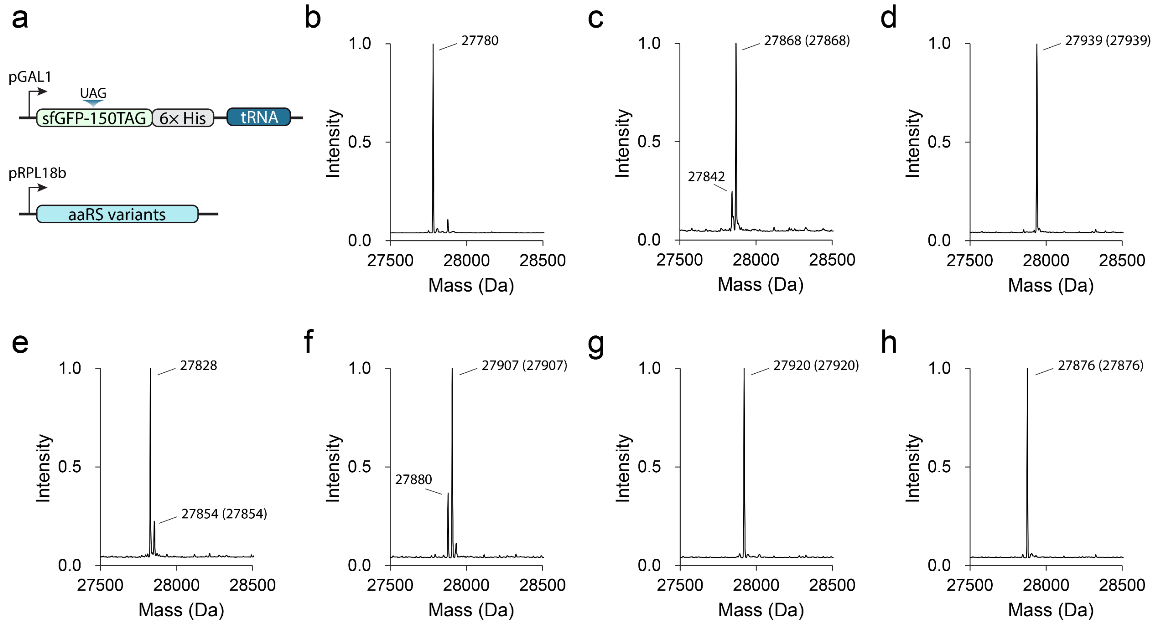

Supplementary Fig. 2. Protein mass spectrometry of sfGFP. (a) Schematic for the plasmids used for sfGFP expression and purification. (b-g) Whole-protein mass spectrometry of WT sfGFP (b) and sfGFP-150ncAA expressed with the top performing aaRS variant clone in each evolution campaign AcFRS/AzMF-1-A (c), AcFRS/IF-C (d), LeuOmeRS/AzF-A (e), *Ma*PylRS/AzK-B (f), *Ma*PylRS/PhK-A (g), and *Ma*PylRS/PrK-A (h) with 1 mM cognate ncAA added. The observed mass for WT sfGFP (27780 Da) was 89 Da lower than the expected mass (27869 Da), suggesting that the first methionine was cleaved and the N-terminus was acetylated in *S. cerevisiae*. Theoretical masses for sfGFP-150TAG proteins containing ncAA were calculated based on the observed mass for WT sfGFP (27780 Da) and written in parentheses. The sfGFP-150ncAA proteins expressed in the presence of AzMF, AzF, and AzK showed a lower peak in addition to the expected peak (c, e, and f), which is presumably due to the instability of the azide group during the ionization process in ESI-MS. Source data are provided as a Source Data file.

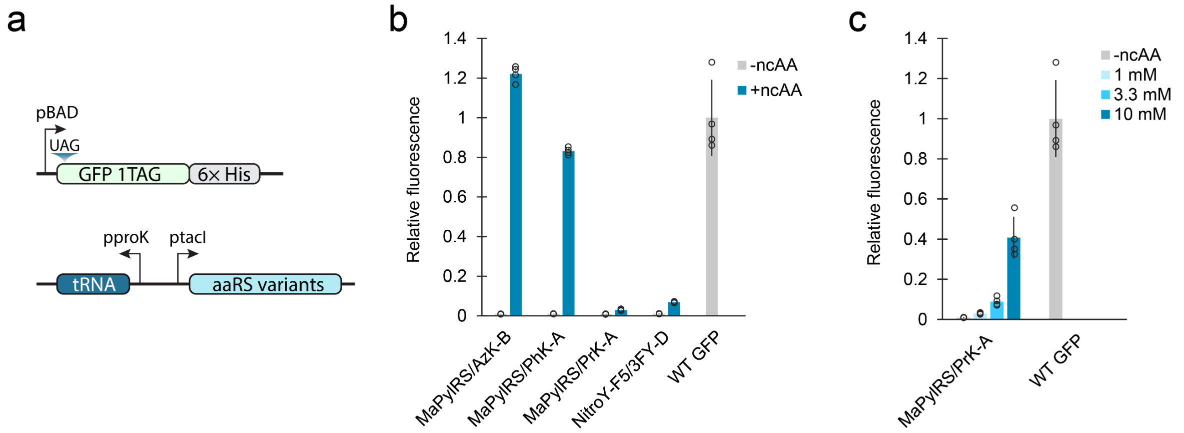

Supplementary Fig. 3. ncAA incorporation by individual evolved PylRS variants in *E. coli* with individual target ncAA. (a) Schematic for the plasmids used for activity measurement in *E. coli*. (b and c) GFP was expressed with plasmids encoding the indicated PylRS variants in the presence or absence of target ncAA (10 mM for 3FY and 1 mM for the other ncAAs in panel (b) and the indicated concentrations of PrK in panel (c)). Fluorescence of GFP with an amber codon was nornalized to that of wild-type GFP (WT GFP). Each condition was measured in biological quadruplicates, and the mean ± one standard deviation (error bars) is shown. Source data are provided as a Source Data file.
