# Supplementary material for: Directed evolution of aminoacyl-tRNA synthetases through in vivo hypermutation
Source: Nat Commun. 2025 May 24;16:4832. doi: 10.1038/s41467-025-60120-w (PMC12103617; doi:10.1038/s41467-025-60120-w)
Supplement: Supplementary file 2 — Description of Additional Supplementary Files [file 41467_2025_60120_MOESM2_ESM.pdf]

Description of Additional Supplementary Files

File Name: Supplementary Data 1
Description: The details of the evolution campaigns performed

File Name: Supplementary Data 2
Description: Mutations for all individual evolved aaRS sequences characterized

File Name: Supplementary Data 3
Description: Fold change in RRE value shown in Figure 2a

File Name: Supplementary Data 4
Description: Fold change in RRE value shown in Figure 3a

File Name: Supplementary Data 5
Description: Plasmids used

File Name: Supplementary Data 6
Description: Reagents used

File Name: Supplementary Data 7
Description: Yeast strains used
